# Systemic long-term metabolic effects of acute non-severe paediatric burn injury

**DOI:** 10.1038/s41598-022-16886-w

**Published:** 2022-07-29

**Authors:** Sofina Begum, Blair Z. Johnson, Aude-Claire Morillon, Rongchang Yang, Sze How Bong, Luke Whiley, Nicola Gray, Vanessa S. Fear, Leila Cuttle, Andrew J. A. Holland, Jeremy K. Nicholson, Fiona M. Wood, Mark W. Fear, Elaine Holmes

**Affiliations:** 1 Harvard Medical School, Harvard University, 25 Shattuck Street, Boston, MA 02115 USA; 2 Channing Division of Network Medicine, Brigham and Women’s Hospital, 181 Longwood Avenue, Boston, MA 02115 USA; 3 Department of Metabolism, Digestion and Reproduction, Faculty of Medicine, Imperial College London, Sir Alexander Fleming Building, South Kensington, London, SW7 2AZ UK; 4 Australian National Phenome Centre, Health Futures Institute, Murdoch University, Harry Perkins Institute, Perth, WA 6150 Australia; 5 School of Biomedical Sciences, The University of Western Australia, Perth, WA Australia; 6 Perron Institute for Neurological and Translational Science, Nedlands, WA Australia; 7 Centre for Computational and Systems Medicine, Health Futures Institute, Murdoch University, Harry Perkins Institute, Perth, WA 6150 Australia; 8 Translational Genetics, Telethon Kids Institute, Perth, WA Australia; 9 Faculty of Health, Centre for Children’s Health Research, School of Biomedical Sciences, Queensland University of Technology (QUT), South Brisbane, QLD Australia; 10 The Children’s Hospital at Westmead Burns Unit, Department of Paediatrics and Child Health, Sydney Medical School, Kids Research Institute, The University of Sydney, Sydney, NSW Australia; 11 Medical School, University of Western Australia, Harry Perkins Institute, Murdoch, Perth, WA 6150 Australia; 12 Faculty of Medicine, Institute of Global Health Innovation, Imperial College London, Level 1, Faculty Building South Kensington Campus, London, SW7 2AZ UK; 13 WA Department of Health, Burns Service of Western Australia, Perth, WA 6150 Australia

**Keywords:** Trauma, Paediatric research, Outcomes research

## Abstract

A growing body of evidence supports the concept of a systemic response to non-severe thermal trauma. This provokes an immunosuppressed state that predisposes paediatric patients to poor recovery and increased risk of secondary morbidity. In this study, to understand the long-term systemic effects of non-severe burns in children, targeted mass spectrometry assays for biogenic amines and tryptophan metabolites were performed on plasma collected from child burn patients at least three years post injury and compared to age and sex matched non-burn (healthy) controls. A panel of 12 metabolites, including urea cycle intermediates, aromatic amino acids and quinolinic acid were present in significantly higher concentrations in children with previous burn injury. Correlation analysis of metabolite levels to previously measured cytokine levels indicated the presence of multiple cytokine-metabolite associations in the burn injury participants that were absent from the healthy controls. These data suggest that there is a sustained immunometabolic imprint of non-severe burn trauma, potentially linked to long-term immune changes that may contribute to the poor long-term health outcomes observed in children after burn injury.

## Introduction

Paediatric burn injury remains one of the most devastating traumas; not only as a result of the acute impact of the primary injury but also the subsequent increased risk of long-term morbidities. This increased risk of morbidity encompasses a range of conditions including cardiovascular disease, respiratory illness and mental health disorders with elevated age-adjusted mortality^1–6^. Long-term systemic changes have previously been reported as a consequence of severe childhood burns^7^, but increasing evidence suggests even non-severe burns may have long-term effects^1–6^. This is important as in many developed countries non-severe injury accounts for over 90% of paediatric burns, whilst burns remain a very common mechanism of injury^8^.

The physiological changes that underpin long-term systemic effects of non-severe burn injury remain undefined, with much still unknown regarding the potential long-term pathological modifications. However, burn trauma associated hypermetabolism has been found to be persistent long after injury irrespective of severity, with perturbed metabolic and systemic inflammatory changes seen in both children and adults^6,9^. These systemic changes have been observed in both severe (> 20% total body surface area, (TBSA)) and non-severe burns. It has been established that in burn induced hypermetabolism, resting energy expenditure is increased two-fold in comparison to normal healthy rates, with increased heart rate, breathing, body temperature, oxygen consumption and carbon dioxide production, associated with sustained hyperglycemia and insulin resistance^9–11^. As a result of burn hypermetabolism, an overall leaner body mass has been observed in burns patients due to the increased metabolic output^9^.

Burn injury is known to induce a chronic inflammatory response that has a substantial impact on multiple organs and systems. Elevated serum and hepatic concentration of proinflammatory cytokines, Interleukin-1α/β (IL-1α/β), Interleukin-6 (IL-6), and Tumour necrosis factor-α (TNF-α), are increased in burn injury patients and contribute to hepatic dysfunction observed in some patients^7^. Whilst much of the current literature on identifying mechanistic pathways associating burn injuries with subsequent pathology has focused on the immune system, metabolic profiling studies have highlighted other dysregulated metabolic pathways, involving hepatic dysfunction, urea cycle perturbation and mitochondrial defects.

Based on prior observations of hypermetabolism and perturbed amino acid metabolism in individuals with prior burn injury^6,7,10,11^, we have used targeted mass spectrometry (MS) based assays for measuring serum amino acids to identify perturbations in metabolism in children with prior burn injury. We also used a targeted MS method for measuring 15 tryptophan pathway metabolites since perturbations in the tryptophan pathway have been observed in children who have suffered thermal injury^12^. Furthermore, we identify correlative patterns between these metabolites and the underlying immune changes by mapping the association between the metabolites and a cytokine panel in these individuals. The data present new insights into potential links between sustained metabolic and immune dysfunction after non-severe burn injury in children.

## Results

**Figure 1:**
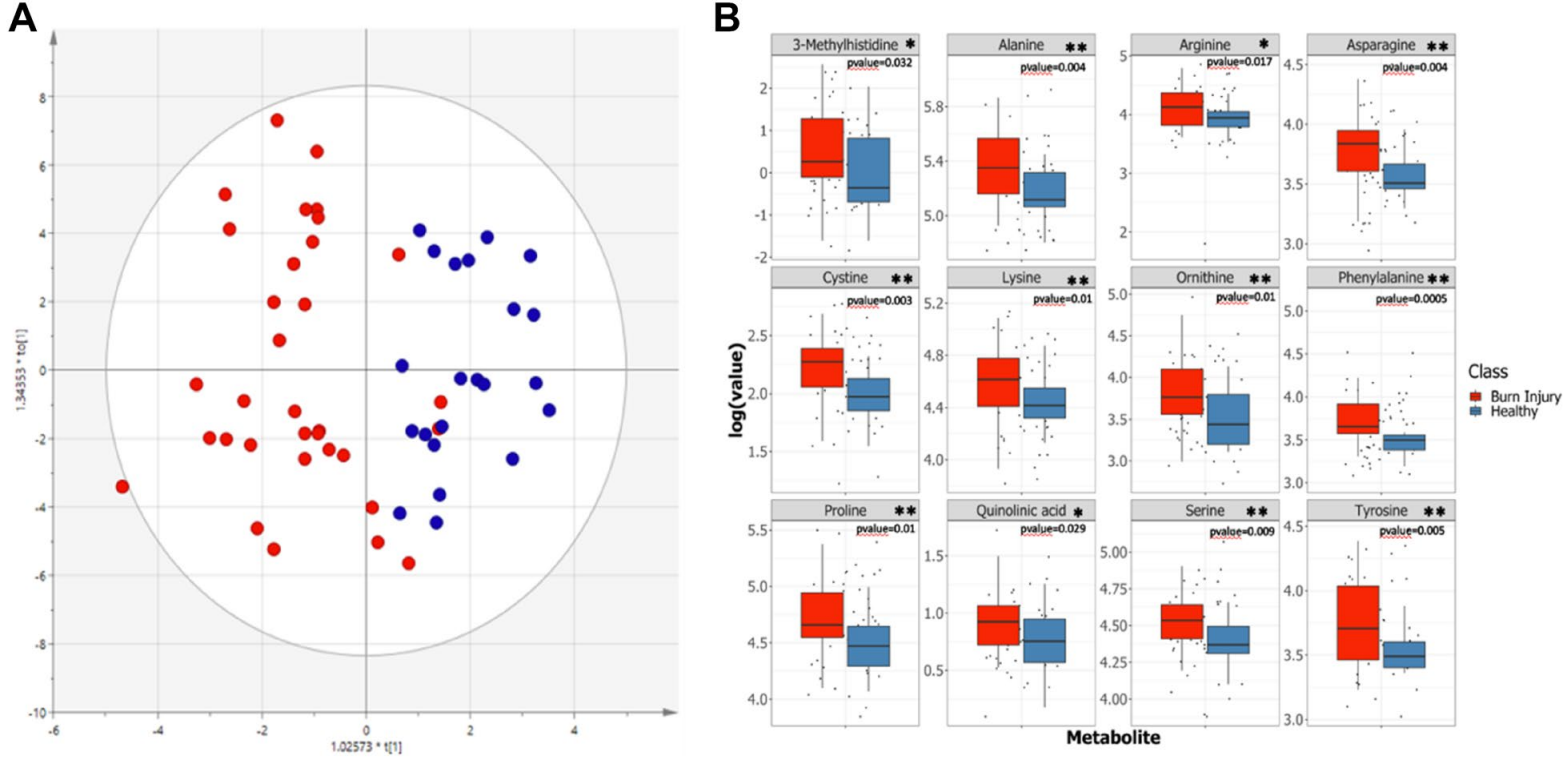
Orthogonal partial least square discriminant analysis (OPLS-DA). (**A**) OPLS-DA for class (burn injury or non-burn control) discrimination based on metabolic differences (R^2^X = 0.524, Q^2^Y = 0.2) (**B**) Log-scaled quantified metabolite concentrations, found to be significantly different between burn injury (n = 33) and non-burn (n = 33 age and gender matched (healthy)) controls (*p* value * < 0.05, ** < 0.01).

Differences in the plasma metabolite panels of prior burn injury three years after the event compared to control participants were explored using Principal Components Analysis (PCA) and Orthogonal Partial Least-Squares Discriminant Analysis (OPLS-DA) to identify global differences between the two classes based on inherent metabolic patterns. The burn-injury cohort had a significantly different plasma metabolite profile compared to the non-burn controls, based on the OPLS-DA model calculated with one orthogonal and one aligned component (R^2^X = 0.524, Q^2^Y = 0.2; Fig. 1A). From the PCA scores plot (Fig. S1A), the plasma samples from burn patients and controls were overlapped to a large extent. However, a subset of the prior burn patients are separated from the controls and remaining burn patients. This subset of individuals with prior burn injury was associated with high levels of branched chain amino acids (leucine, isoleucine, valine), tyrosine, methionine and lysine. (Fig. S1B). The OPLS-DA model showed good separation of the two groups with the exception of three samples from the burn group, which were overlapped with the controls (Fig. 1). In order to assess the robustness of the models, the cross validated OPLS-DA scores were plotted and showed that 17 of the 30 prior burn injury patients were not overlapped with the control population (Fig. S2). Additionally, the AUROC was calculated and found to be 0.97 for the burn injury group. Permutation testing and calculation of the cross validated OPLS-DA scores plot indicated that the metabolic differentiation between the control and burn injury group was robust (Fig. S2). Based on the variable importance of projection (VIP) plot (Fig. S5 & Table S1), 22 of the 46 measured metabolites were found to contribute to the differential metabolite pattern associated with burn injury, all of which were elevated in the burn injury group.

In order to identify which individual metabolites were significantly different between the burn and control groups, the Wilcoxon rank sum testing was applied to each variable using Benjamini–Hochberg correction to adjust for multiple testing. A total of 12 of the 46 measured metabolites were found to be statistically significant between burn injury and control groups (Fig. 1B, Table 1), of which 10 overlapped with the differential metabolites identified from the multivariate analysis based on VIP score and rank.

**Table 1:** Ranked univariate testing on all metabolites between burn Injury and non-burn (healthy) controls.

Metabolite	Wilcoxon Rank Sum	Fold Change (logFC)	VIP score (rank)
Phenylalanine	0.0005	0.0549	0.75 (31)
Cystine	0.0035	0.0921	1.03 (21)
Asparagine	0.0041	0.0952	1.48 (4)
Alanine	0.0042	0.0968	1.52 (2)
Serine	0.0092	0.0645	1.29 (8)
Proline	0.01	0.1111	1.37 (5)
Lysine	0.016	0.0732	1.52 (1)
Arginine	0.017	0.1037	1.25 (12)
Ornithine	0.018	0.1304	1.24 (11)
Tyrosine	0.028	0.1091	1.50 (3)
Quinolinic acid	0.029	0.0812	1.01 (22)
3-Methylhistidine	0.032	0.2428	1.05 (19)

Key differential metabolites included phenylalanine, cystine, asparagine, alanine, serine, proline, lysine, arginine, ornithine, tyrosine, quinolinic acid and 3-methylhistidine, Branched chain amino acids (BCAAs) were influential in the OPLS-DA model but did not retain significance in the Wilcoxon sum rank test after Benjamini–Hochberg correction.

A metabolite-metabolite correlation matrix, calculated separately for the burn injury and control groups and hierarchically clustered according to the distribution for the burn injury group, showed stronger inter-metabolite association in the burn group. For the burn group, five main clusters of positively correlated metabolites relating to (A) Branched chain amino acids (BCAAs) and aromatic amino acids along with lysine, alanine, ornithine, methionine, serine and proline; (B) tryptophan pathway metabolites, (C) glutamate, aspartate and arginine, (D) cystine, taurine, ethanolamine and serotonin, and (E) sarcosine and histidine were identified (Fig. S3). The healthy controls showed a more disordered pattern although some correlation between the BCAAs (cluster D) and some of the tryptophan pathway metabolites was apparent.

**Figure 2:**
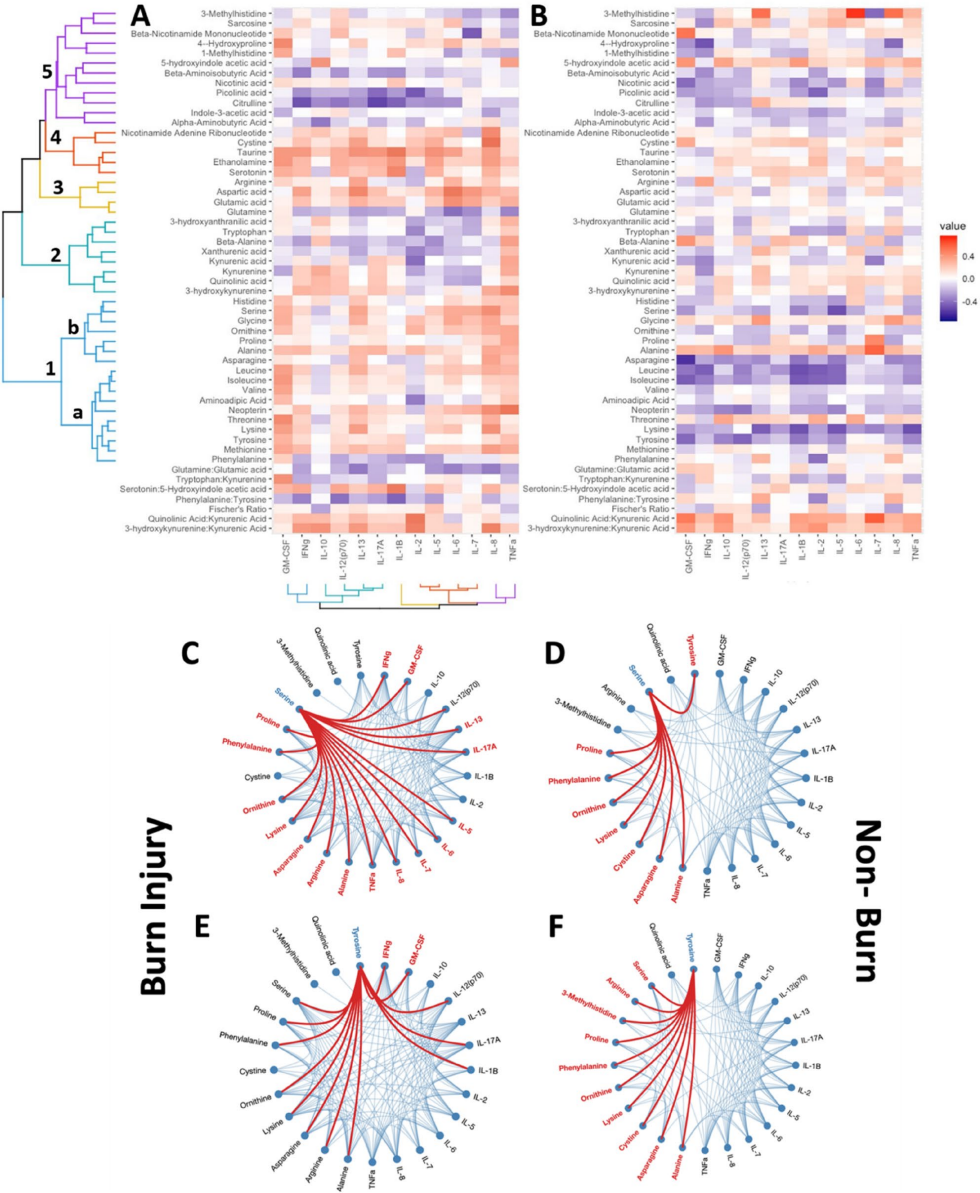
Correlation heatmaps integrating biogenic amines and tryptophan pathway metabolite concentrations and circulating cytokines. (**A**) Measurements from burn injury patients. (**B**) Measurements from non-burn (healthy) controls. Hierarchical clustering based on clustering from burn injury results and mirrored for direct comparison to non-burn (healthy) age and sex matched controls. Hierarchical edge clustering based on correlation analysis of significant metabolites (*p* value < 0.05) against cytokine measurements, visualised as (**C**) burn injury and (**D**) non-burn (healthy) controls focused on positive serine-cytokine interactions, and (**E**) burn injury and (**F**) non-burn (healthy) controls focused on positive tyrosine-cytokine interactions.

To interrogate the association between the significant metabolic and immune changes post-burn correlational analysis of metabolites was performed with cytokine data previously reported from this cohort^10^. As with the metabolite-metabolite correlations, generally metabolite-cytokine correlations were stronger in the burn injury group than the control group (Fig. 2), with 22 significant metabolite-cytokine correlations in non-burn controls, and 50 significant metabolite-cytokine correlations in the burn injury group (*p* value < 0.05) (Supplementary Tables S3 and S4) . With the exception of 3-methylhistidine and quinolinic acid, all the significantly differentially detected metabolites showed a positive correlation with Granulocyte macrophage colony stimulating factor (GM-CSF), TNF-α, Interleukin-8 (IL-8), and Interleukin-13 (IL-13), in the burn injury group and weaker positive correlations with Interferon-gamma (IFN-γ), IL-13 and Interleukin-17A (IL-17A). Although the general trend was towards a positive correlation between most metabolites and cytokines, Interleukin-2 (IL-2), and Interleukin-10 (IL-10)did not follow the same pattern (Fig. 2A,C). For the control group, the BCAAs, aromatic amino acids, lysine, serine and arginine demonstrated generally negative associations with most cytokines except IL-6 (Fig. 2B,D).

**Figure 3:**
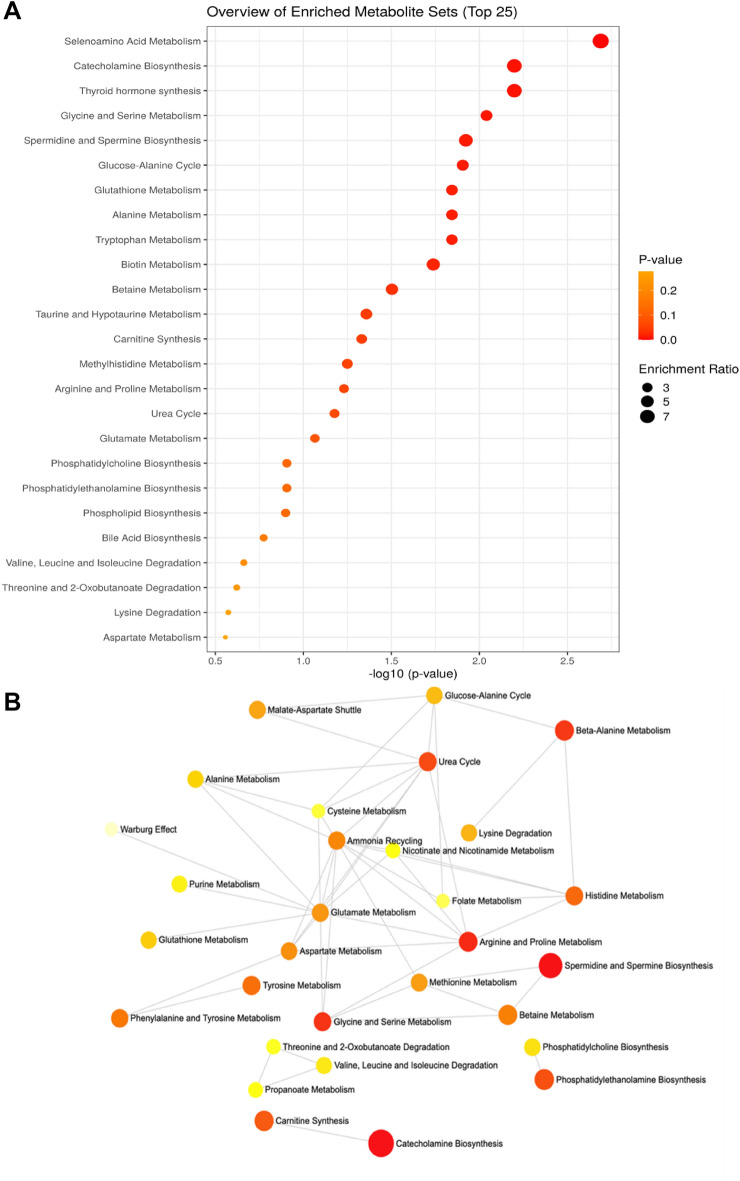
Quantitative enrichment analysis based on all 46 metabolites, discriminating burn injury and healthy controls. (**A**) Overview of the top 25 enriched metabolite sets and (**B**) corresponding metabolite network of the same top 25 enriched metabolite sets.

To further illustrate the difference between the control and burn groups, the interaction of two metabolites (tyrosine and serine), which showed contrasting relationships with cytokines were modelled using hierarchical edge clustering, linking significant metabolites (*p* value < 0.05) with cytokines (Fig. 2C,D), highlighting the difference in immune-metabolite interactions between cohorts. Whereas no metabolite-cytokine correlations above (r = 0.8) existed in the control group using the hierarchical edge bundling technique, the burn-injury group showed a range of correlations above this level including those between the metabolite hubs serine and tyrosine and IFN-γ, GM-CSF, Interleukin-12 (IL-12 (Fig. 2)). This suggests disruption in liver metabolism post burn injury and highlights the correlation between liver and immune function, further supported by pathway enrichment analysis (Fig. 3). Quantitative enrichment analysis using all 46 metabolites for broader pathway enrichment, highlighted multiple significant pathways including catecholamine biosynthesis and amino acid metabolic pathways (Fig. 3A,B) (Supplementary Table S5) that discriminated between the burn and control groups.

Notably, urea cycle, ammonia recycling and arginine and proline metabolism form a strong triad in the network fed by the malate-aspartate shuttle emphasizing the upregulated amino acid metabolism and increased detoxification via urea excretion.

## Discussion

The changes in levels of metabolites observed in this cohort were consistent with previous studies demonstrating a significant increase in the turnover of arginine, ornithine, proline and leucine that appears specific to post-burn injury^13^. Alanine and increased levels of plasma aromatic amino acids have also been associated with burn injury but are altered after other trauma types, suggesting these changes may be associated with a more general stress response^14^.

Increased serum concentrations of aromatic amino acids, alanine and lysine have been associated with liver disease^15^. The Fischer’s ratio (branched-chain amino acids to aromatic amino acids) is used as a diagnostic marker for assessing liver metabolism, hepatic functional reserve, and the severity of liver dysfunction, with a decreased ratio indicative of dysfunction^16^. Here we observed a trend towards reduced Fischer’s ratio in the burn injury cohort (Supplementary Table S2). Whilst non-significant, the trend is consistent with the previously reported prolonged insult to hepatic function following severe burn injury and associated with a profound hypermetabolic and hyperinflammatory response^7^. This suggests that even non-severe burn injury may have a profound and lasting effect on the liver in children.

At a population level it has been established that burn injury (severe and non-severe) contributes to long-term risk of liver disease in a dose-dependent manner, based on hospital admissions^17^. In tandem with our data this suggests non-severe burn injury may disrupt normal liver function at a sub-clinical level, posing a chronic risk of morbidity. Our results are consistent with other studies that have shown similarly long “memory” in the liver and muscle tissues following severe burn injury with lysine, urea cycle and tryptophan metabolism being amongst the most disrupted pathways^18^.

Disturbances in the tryptophan pathway have previously been implicated in severe burn injury in children, with increased urinary kynurenine observed in burn injury patients^12^. This pathway has potentially toxic downstream metabolites, most notably quinolinic acid, which is a known neurotoxin, and has been reported to be elevated in several neuropathological conditions including Alzheimer’s Disease^19^ and juvenile idiopathic inflammatory myopathy^20^. The neuropathophysiological effect of quinolinic acid synthesis have been linked to major depressive disorder, where increased concentrations were observed in blood, cerebrospinal fluid, and brain tissue^21^.

Depression and other mental health disorders are a common morbidity for burn survivors^22^, have a significant impact on quality of life and maybe linked to these elevated levels of quinolinic acid. Dantzer et al.proposed that the perturbation of tryptophan metabolism was a result of chronic inflammatory signalling, representing a major contributor of the pathogenesis of depressive disorders and that the neurotoxic effects of quinolinic acid were mediated via lipid peroxidation and disruption of the blood–brain barrier^23,24^. Saito et al. showed that quinolinic acid was elevated in plasma and cerebrospinal fluid after systemic immune stimulation and that this was mediated by indoleamine dioxygenase (IDO), which catalyses the first step of the tryptophan-kynurenine metabolic pathway^25^. The expression of IDO is upregulated in response to signalling by inflammatory cytokines including interferons (IFN-α, -β, and -γ) and TNF-α^24^. Previously reported cytokine data from this cohort demonstrated elevated levels of IFN-γ and TNF-α^26^. However, in the current study, although quinolinic acid, IFN-γ and TNF-α serum levels were all higher in the burn patients, the correlation between quinolinic acid and these cytokines was weak.

Burn-injury has been associated with an increased risk of type 2 diabetes. In one study, the risk of being admitted for type. 2 diabetes was 2.21 times higher in individuals who had suffered a burn injury in the previous 5 years^27^. In a study in children, serum amino acid concentrations were found to correlate with insulin resistance^28^. In general elevated serum levels of amino acids, particularly BCAAs, aromatic amino acids, alanine, glutamate, ornithine and lysine, are associated with worse metabolic health, including risk of future type 2 diabetes and were predictive of developing type 2 diabetes in a five-year follow-up study assessing metabolic health in a general population^29^. In the current study all of these metabolites, with the notable exception of glutamate, were found to be significantly higher in the burn injury group either in the Wilcoxon rank sum (alanine, ornithine, lysine, phenylalanine and tyrosine) or the OPLS-DA (all except phenylalanine) models. These metabolites are all found in Cluster A (Fig. S3), indicating that they are highly correlated in terms of response to burn injury.

The BCAAs and aromatic amino acids were the key metabolic drivers for the separation of the subgroup of prior burn injury individuals that were clearly differentiated from the control group (Fig. S1A). BCAAs have been shown to impair intracellular insulin signalling and glycogen synthesis^30^ and are known to induce an insulin response via direct stimulation of the pancreatic beta cells and activation of the mammalian target of rapamycin (mTOR) mitogenic signalling pathway^31^. The literature points to a shift from the acute post burn Th1 to chronic Th2 response with Th2 cytokines such as GM-CSF, TNF-α, IL-2 and IL-17 remaining elevated for at least 3 years after the initial burn event^7^. Given the association of BCAAs and aromatic amino acids with type 2 diabetes, the current results showing elevation in individuals with prior burn injury, and their correlation to a range of proinflammatory cytokines, warrants further exploration of the association between the branched and aromatic acids and the Th2 inflammatory response in longitudinal burn studies.

Other amino acids, such as serine, showed strong correlations with other metabolites and cytokines only in the burn injury patients and not in controls. Serine and glycine were closely clustered and showed similar behaviour in the burn injury patients in contrast to the control patients. In participants with prior burn injury serine and glycine correlated strongly with TNF-α and IL-8, again supporting a link between metabolic and immune changes after non-severe burn.

The general increase in the strength of metabolite cytokine correlations has been documented previously in rat models following infection inducing both Th1 and Th2 inflammatory responses^32^ and is consistent with the finding of correlation between the chronic low-level pro-inflammatory milieu that persists after a non-severe burn and the broad panel of metabolic changes.

The enrichment analysis highlighted a number of pathways that may be altered in the burn cohort, including the catecholamine synthesis pathway. This is consistent with findings in severe burn patients, where norepinephrine and epinephrine titres are elevated for prolonged periods following injury^7^. Disruption of catecholamine regulation may be relevant to the observed increased cardiovascular risk post-burn, as elevated catecholamines are thought to contribute to myocardial hypertrophy and endothelial inflammation through increased heart rate and blood pressure^33^. Prolonged cardiovascular stress has previously been described in severe burn patients^34^ whilst antagonising catecholamines in a mouse model of non-severe burn injury ameliorated myocardial hypertrophy^35^. Platelet aggregation is enhanced by stimulation of adrenergic receptors, which in combination with endothelial dysfunction may contribute to atherothrombosis, myocardial infarction^36^, and other circulatory system diseases increased in the years after non-severe pediatric burn^2^ that may be linked to some of the metabolic changes observed in this study.

Ammonia recycling, arginine and proline metabolism and the urea cycle form key connected hubs in the metabolite enrichment network from this data. This highlights the importance of the urea cycle in detoxification of the likely upregulated muscle and mitochondrial metabolism that has been observed after severe burns, and by connection to the glucose-alanine cycle (Cahill cycle) and the malate-aspartate shuttle (Borst cycle) respectively and supports further study into the impacts of burn on these pathways.

Whilst the data presented offer new insights into dysregulated metabolism post burn injury, the limitations of the study lie mainly in the low number of burn patient and control samples, as well as the use of a single centre cohort. Whilst the cohort recruited reflects the etiology of burn in the age group tested, with scald injuries dominant, the small numbers in each group restricted the ability to investigate whether factors such as age, gender or the etiology of injury were important or linked to the metabolic or immune changes observed. Therefore in this study we were not able to account for the possible impact of these factors. Further studies in larger and multi-centre settings will be important to validate the findings here and will likely shed light on whether etiology, age, gender or TBSA affect the metabolic changes observed in this study. Additionally, the plasma metabolome presents one side of the total picture, which could be complemented by co-analysis of urine and fecal metabolic profiles, which were not measured in the current study. Nevertheless, despite these limitations, the results are valuable in expanding our understanding of the persistent metabolic impacts of burn injury in children since the majority of burn injury research has focussed on adult cohorts.

In summary, this data provide evidence for an “immunometabolic memory” of non-severe burn injury in children for over three years. These sustained changes are concordant with a role in the observed immune dysfunction and long-term morbidity identified in burn patients, although this requires further research to validate a link. Increasingly acute burn childhood trauma appears to be linked to sustained systemic impacts and long-term morbidity. Further investigation to better understand the drivers of this change and possible options for clinical intervention is required to improve the long-term health outcomes of children with a burn.

## Materials and methods

### Study design and sample collection

Children (n = 36) were recruited at least 3 years after presenting for a non-severe burn injury at Princess Margaret Hospital with the voluntary informed consent of a parent or guardian. All children were aged 0–4 years of age at the time of original presentation for the burn injury and were aged between 4 and 8 years old at time of sample collected. Age/sex-matched controls were selected from a pool of healthy donors. All samples were obtained with informed consent and the collection conducted with ethical approval from the Child and Adolescent Health Service WA (approval numbers: 2015219EP; 1111EP; 768EP). All work was performed in accordance with the relevant guidelines and regulations and performed in accordance with the declaration of Helsinki. All patients recruited had no history of pre-existing illness and were not currently on medication at the time of sampling. No patients had visible signs or recent history of acute infection at the time of blood collection.

Mean total body surface area (TBSA) was 4 ± 3.2%, mean age at time of injury was 19.2 months ± 9 months and mean age at time of sample collection was 5.8 years ± 0.85 years (Supplementary Table S6). Of the 36 samples collected, Fifteen female and 18 male burn survivors provided sufficient plasma samples for analysis with three samples not sufficient for further study (n = 33 for analysis). Previous analysis using this cohort showed a diminished immune response in the burn injury survivors in comparison to their healthy uninjured counterparts^26^.

### Patient samples and cytokine data

Simultaneous analysis of cytokine and chemokine levels was performed using a custom Milliplex MAP human high sensitivity T cell panel multiplex bead assay (MERCK). Quantified cytokines were Tumour necrosis factor- alpha (TNF-α), Interleukin-8 (IL-8), Interleukin 7 (IL-7), Interleukin-6 (IL-6), Interleukin-5 (IL-5), Interleukin-2 (IL-2), Interleukin-1beta (IL-1β), Interleukin-17A (IL-17A), Interleukin-13 (IL-13), Interleukin-12 p70 (IL-12(p70)), Interleukin-10 (IL-10), Interferon-gamma (IFN-γ) and Granulocyte macrophage colony stimulating factor (GM-CSF), with results as previously reported^26^.

### Metabolite quantification

We applied a metabolic phenotyping approach^37^ to investigate inherent differences in metabolism between children with and without prior burn injury. Validated targeted UHPLC-MS (Ultra High-Performance Liquid Chromatography-Mass Spectrometry) assays were used to quantify 46 biogenic amines, amino acids and tryptophan pathway metabolites^38,39^ in plasma. Lower limit of quantitation (LLOQ) values for metabolites are listed in Supplementary Table S7.

The quantification of biogenic amines and amino acids was executed according to a previously published and validated method^38^ with minor modifications. In brief, 31 amino acids were quantified from 10 µL of plasma. Automated sample extraction was performed using a Biomek i5 sample automation system (Beckman Coulter, Mount Waverley, Victoria 3149, Australia). Samples were diluted 1:1 with water, to which 20 µL of stable isotope labelled (SIL) internal standards in water were added, and protein precipitation was performed by the addition of 90 µL of methanol. Following mixing and centrifugation, 10 µL supernatant was transferred into a Waters 700 µL 96-well plate for derivatization with AccQTag™ reagent (Waters Corp., Milford, MA, USA). The subsequent derivatized samples were diluted 1:4 (v/v) with water prior to Liquid chromatography-Mass spectrometry (LC–MS) analysis.

LC–MS analysis was performed using a Waters Acquity UPLC® coupled to a Xevo TQ-XS mass spectrometer (Waters Corp., Milford, MA, USA). Raw data were then pre-processed for peak integrations and the calculation of metabolite concentrations using the TargetLynx package in MassLynx v4.2 (Waters Corp., Milford, MA, USA).

The quantification of tryptophan and 14 products within the same metabolic pathway was performed as previously reported^39^. In brief, tryptophan metabolites were quantified from 50 µL of plasma. Automated sample extraction was performed using a Biomek i5 sample automation system. SIL internal standards (20 µL) were added to all samples prior to protein precipitation via the addition of 250 µL of methanol containing 2 mM ammonium formate. Following mixing, samples were then transferred to a Phenomenex PHREE™ phospholipid removal solid phase extraction plate (Phenomenex, NSW, Aus). Following washing of PHREE plates with 150 µL of methanol containing 2 mM ammonium formate, eluent collection plates were dried using a SpeedVac vacuum concentrator (Thermo Fisher, Massachusetts, USA). Extracts were re-suspended in 100 µL of water with 0.1% formic acid prior to LC–MS analysis.

LC–MS analysis was performed using a Waters Acquity UPLC® (Waters Corp., Milford, MA, USA) coupled to a Waters Xevo TQ-S MS (Waters Corp., Wilmslow, UK). Raw data were then pre-processed for peak integrations and the calculation of metabolite concentrations using the TargetLynx package in MassLynx v4.2.

### Statistical analysis and data integration

Metabolite quantification data were scaled to account for the 1:1 dilution of sample with water and converted to appropriate scales across both assays. Data were then modelled using principal components analysis (PCA) (Simca V.15.0., Umetrics, Sweden) to investigate variation. Scores plots were used to examine the relationship between controls and burn injury patients. To further interrogate the relationship and differences between controls and burn injury patients, orthogonal partial least-squares discriminant analysis (OPLS-DA) was trained with 1 predictive + 1 orthogonal component. To test the robustness of the model, subsequent statistical analysis was conducted using *R*; with “*ggplot2*” to visualize log-transformed metabolite concentrations. Statistical significance of individual metabolites was evaluated using Wilcoxon Rank Sum testing, corrected for multiple testing (Benjamini-Hochberg, *p* value < 0.01).

Spearman’s correlation was used to interrogate the relationship between each dataset, which were hierarchically clustered and mapped using “ggplot2” visualizations. Ratios of significance for biological interpretation were tested for significance using Wilcoxon Rank Sum testing, corrected for multiple testing (Benjamini-Hochberg, *p *value < 0.01). Spearman’s correlation was conducted to correlate significant metabolites against cytokines. Hierarchical edge bundling for the correlation maps was conducted using R package “edgebundleR”, to interrogate the relationship between significant metabolites (*p* value < 0.05) and cytokines between burn injury and non-burn controls. Quantitative metabolite set enrichment was conducted using the R package “MetaboAnalyst” (version 5.0), enriched against the Small Molecular Pathway Database (smpdb.ca).

## Data availability 

Mass spectrometry datasets related to this article can be found on the European Bioinformatics Institute (EBI) MetaboLights database under the project ID MTBLS2644.

## Supplementary Information

Supplementary Information.
