# Supplementary material for: Systemic long-term metabolic effects of acute non-severe paediatric burn injury
Source: Sci Rep. 2022 Jul 29;12:13043. doi: 10.1038/s41598-022-16886-w (PMC9338081; doi:10.1038/s41598-022-16886-w)
Supplement: Supplementary file 1 — Supplementary Information. [file 41598_2022_16886_MOESM1_ESM.docx]

**Systemic long-term metabolic effects of acute non-severe paediatric burn injury**

**Sofina Begum^1,2,3,4^, Blair Z. Johnson^5^, Aude-Claire Morillon^4^, Rongchang Yang^4^, Sze How Bong^4^, Luke Whiley^4,6,7^, Nicola Gray^4,7^, Vanessa S. Fear^8^, Leila Cuttle^9^, Andrew J.A. Holland^10^, Jeremy K. Nicholson^4,7,11,12^, Fiona M. Wood^5,13^, Mark W. Fear^5*^ and Elaine Holmes^3,4,7*^**

1 Harvard Medical School, Harvard University, 25 Shattuck Street, Boston, Massachusetts, 02115, United States of America.

2 Channing Division of Network Medicine, Brigham and Women’s Hospital, 181 Longwood Avenue, Boston, Massachusetts, 02115, United States of America.

3 Department of Metabolism, Digestion and Reproduction, Faculty of Medicine, Imperial College London, Sir Alexander Fleming Building, South Kensington, London, SW7 2AZ, United Kingdom.

4 Australian National Phenome Centre, Computational and Systems Medicine, Health Futures Institute, Murdoch University, Harry Perkins Building, Perth, Western Australia, 6150, Australia

5 School of Biomedical Sciences, The University of Western Australia, Perth, WA, Australia

6 Perron Institute for Neurological and Translational Science, Nedlands, WA, Australia.

7 Centre for Computational and Systems Medicine, Health Futures Institute, Murdoch University, Harry Perkins Building, Perth, Western Australia, 6150, Australia.

8 Translational Genetics, Telethon Kids Institute, Perth, WA, Australia

9 Queensland University of Technology (QUT), School of Biomedical Sciences, Faculty of Health, Centre for Children’s Health Research, South Brisbane, Queensland, Australia

10 The Children’s Hospital at Westmead Burns Unit, Kids Research Institute, Department of Paediatrics and Child Health, Sydney Medical School, The University of Sydney, NSW, Australia.

11 Medical School, Faculty of Health and Medical Sciences, University of Western Australia, and Department of Endocrinology and Diabetes, Fiona Stanley Hospital, Harry Perkins Building, Murdoch, Perth, WA, 6150, Australia.

12 Institute of Global Health Innovation, Faculty of Medicine, Imperial College London, Level 1, Faculty Building South Kensington Campus, London SW7 2AZ, United Kingdom.

13 Burns Service of Western Australia, WA Department of Health, Perth, Western Australia, 6150, Australia.

*Corresponding authors

**Supplementary Figures:**


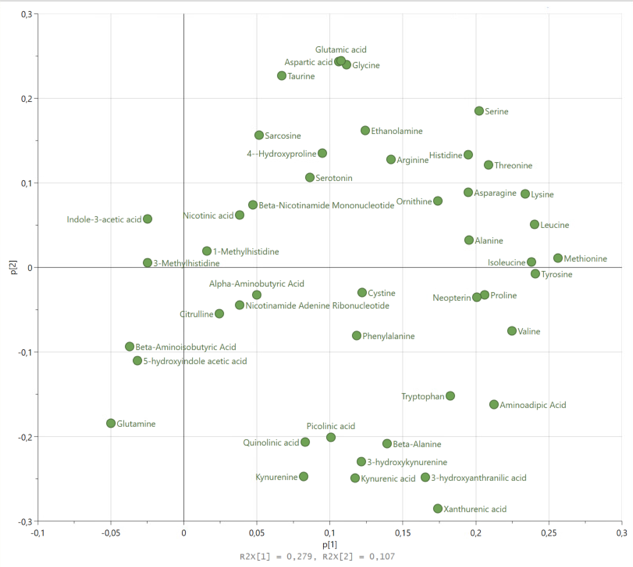


D


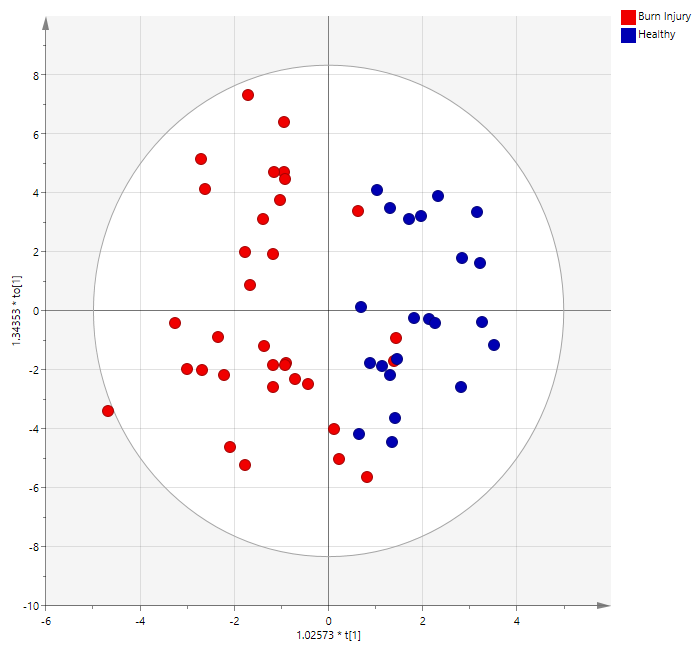

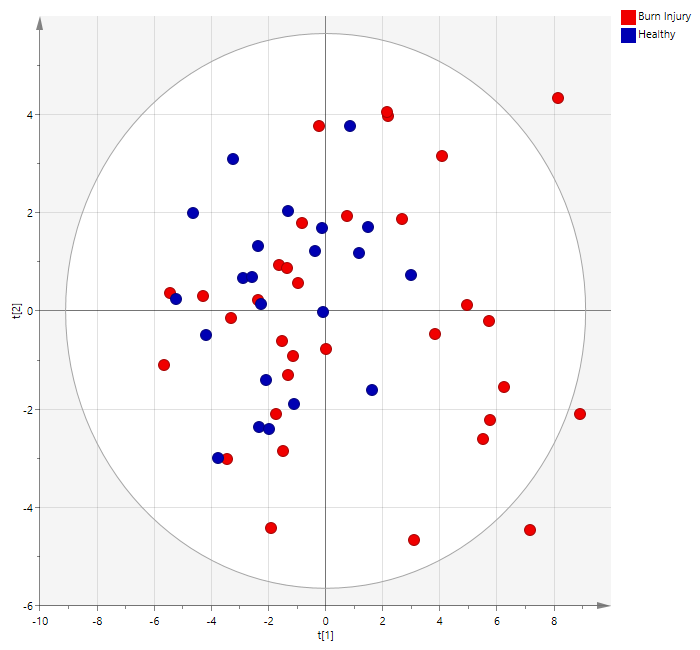

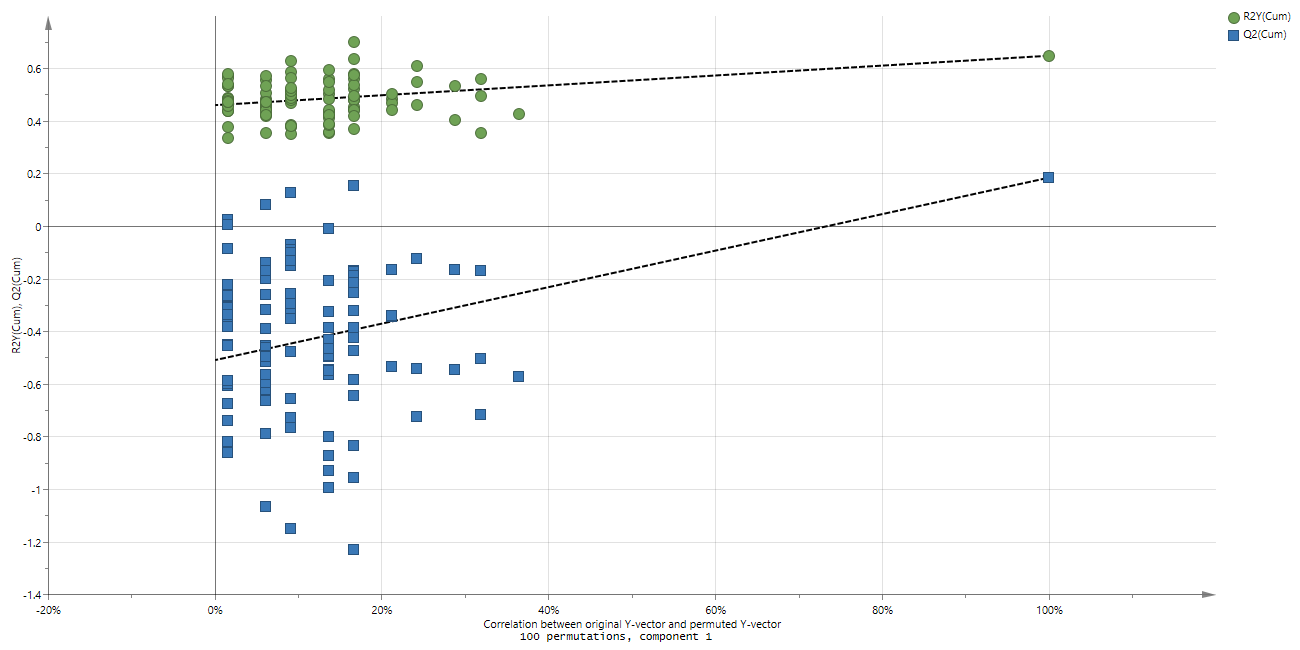


A

B

C

**Figure S1:** Multivariate analysis of quantified biogenic amine and tryptophan pathway metabolites. A) PCA scores plot based on all metabolites. B) PCA loadings plot. C) OPLS-DA for class (burn injury or non-burn healthy control) discrimination based on metabolic differences. C) Permutation testing on OPLS-DA model based on 100 random permutations


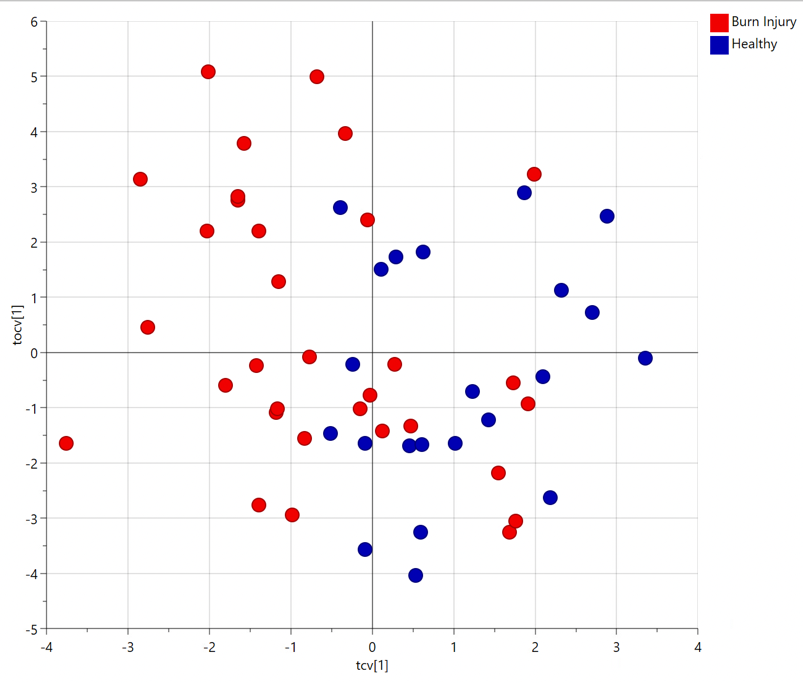

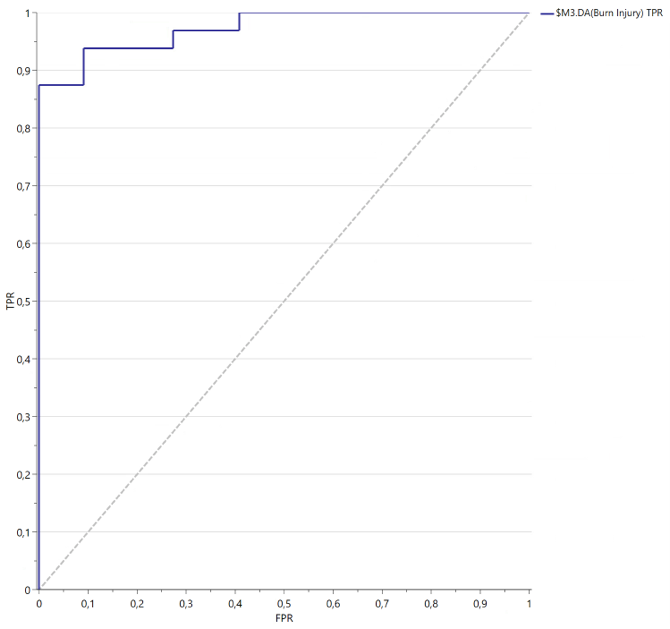


**A**

**B**

**Figure S2:** Multivariate analysis of quantified biogenic amine and tryptophan pathway metabolites. A) Cross validated OPLS-DA for class (burn injury or non-burn healthy control) discrimination based on metabolic differences. B) Area under the curve (AUROC) for OPLS-DA model showing good predictivity (0.97) of prior burns participants.


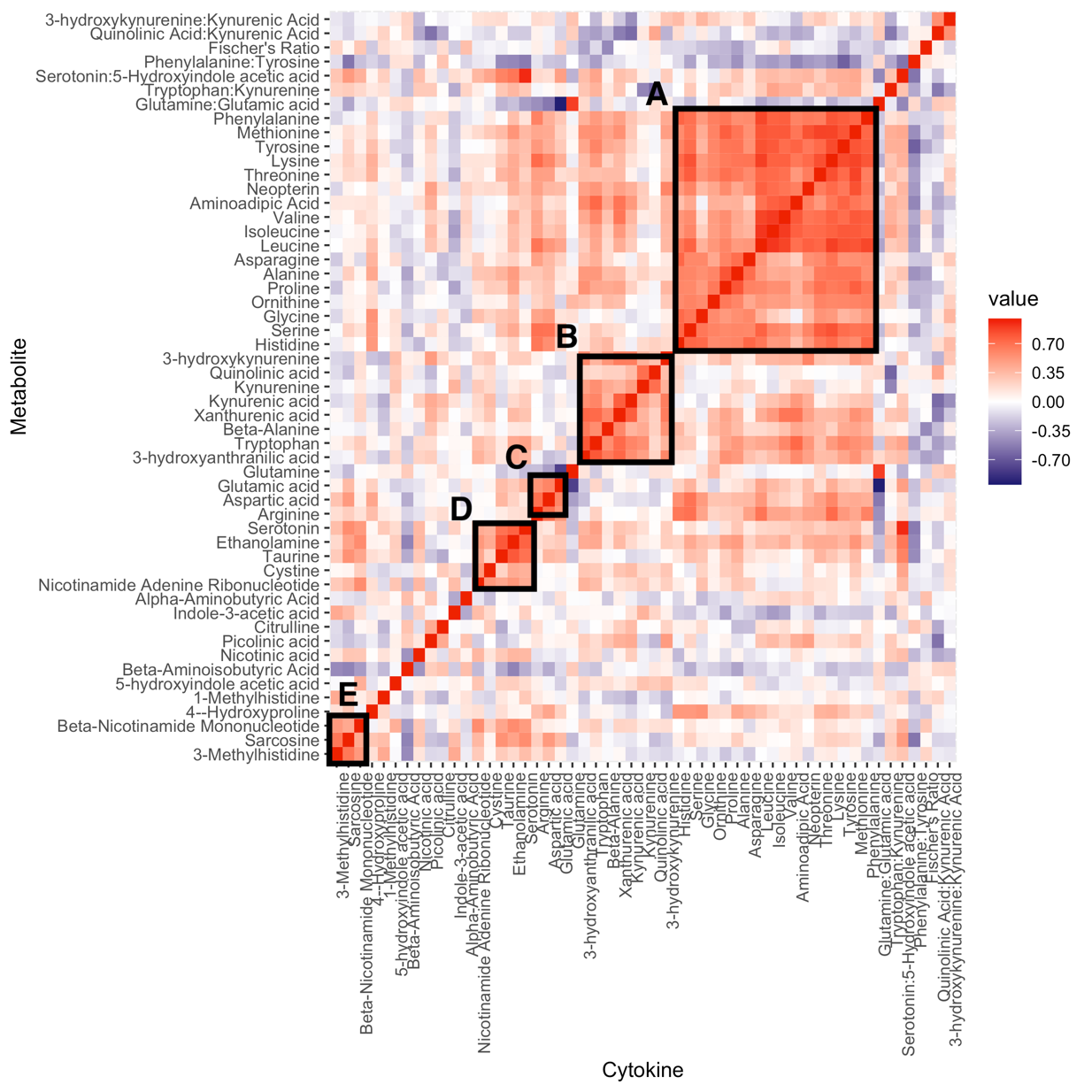


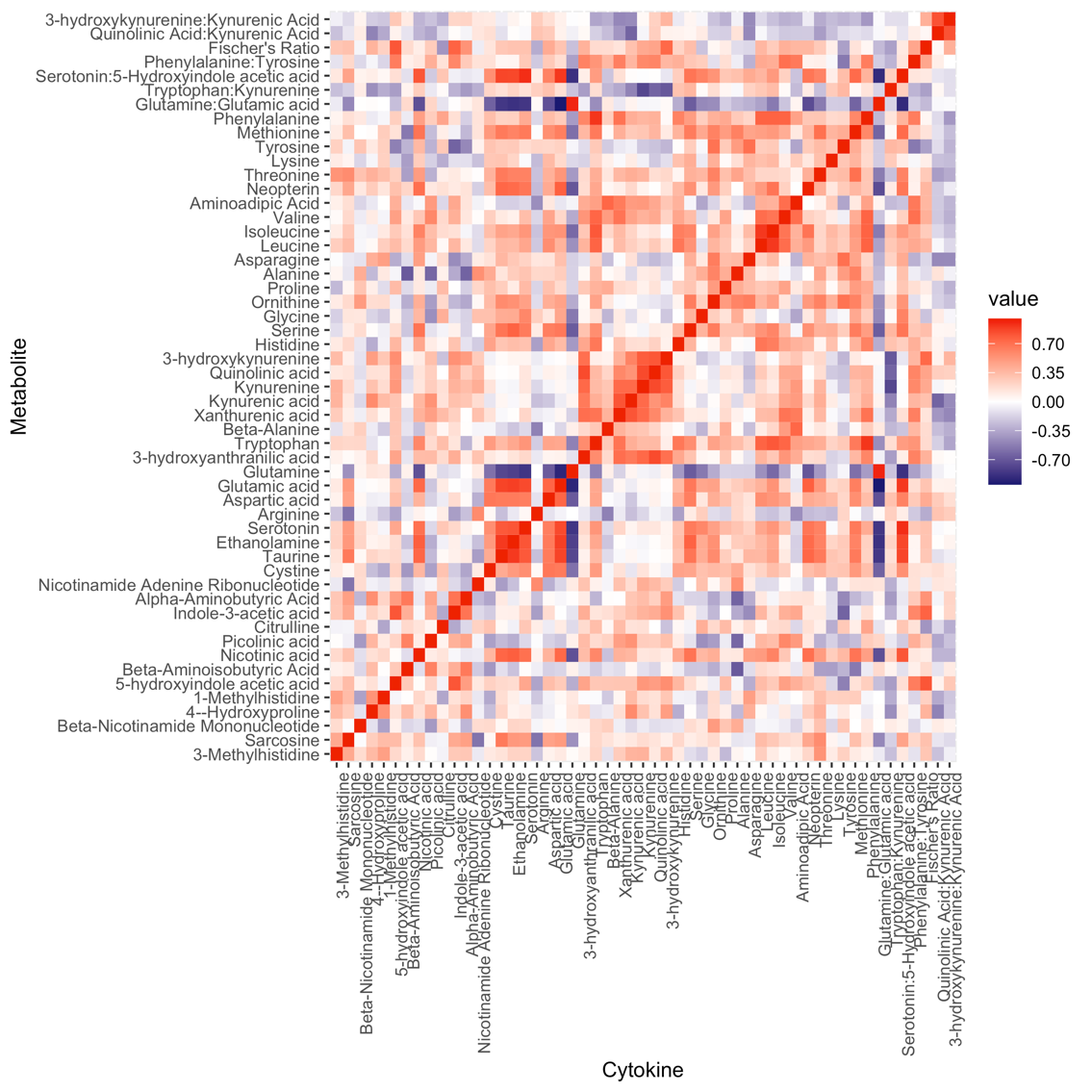


**Metabolite**

**A**

**Metabolite**

**B**

**Figure S3:** Metabolite-metabolite correlation matrix calculated using Spearman’s correlation for A) burn injury participants and B) Healthy participants ordered according to hierarchical clustering of the burn injury group.

**Figure S4:** All 46 quantified metabolites, coloured for burn (red), non-burn healthy controls (blue), pvalue * <0.05** <0.01, corrected for multiple testing using the Bonferroni meth
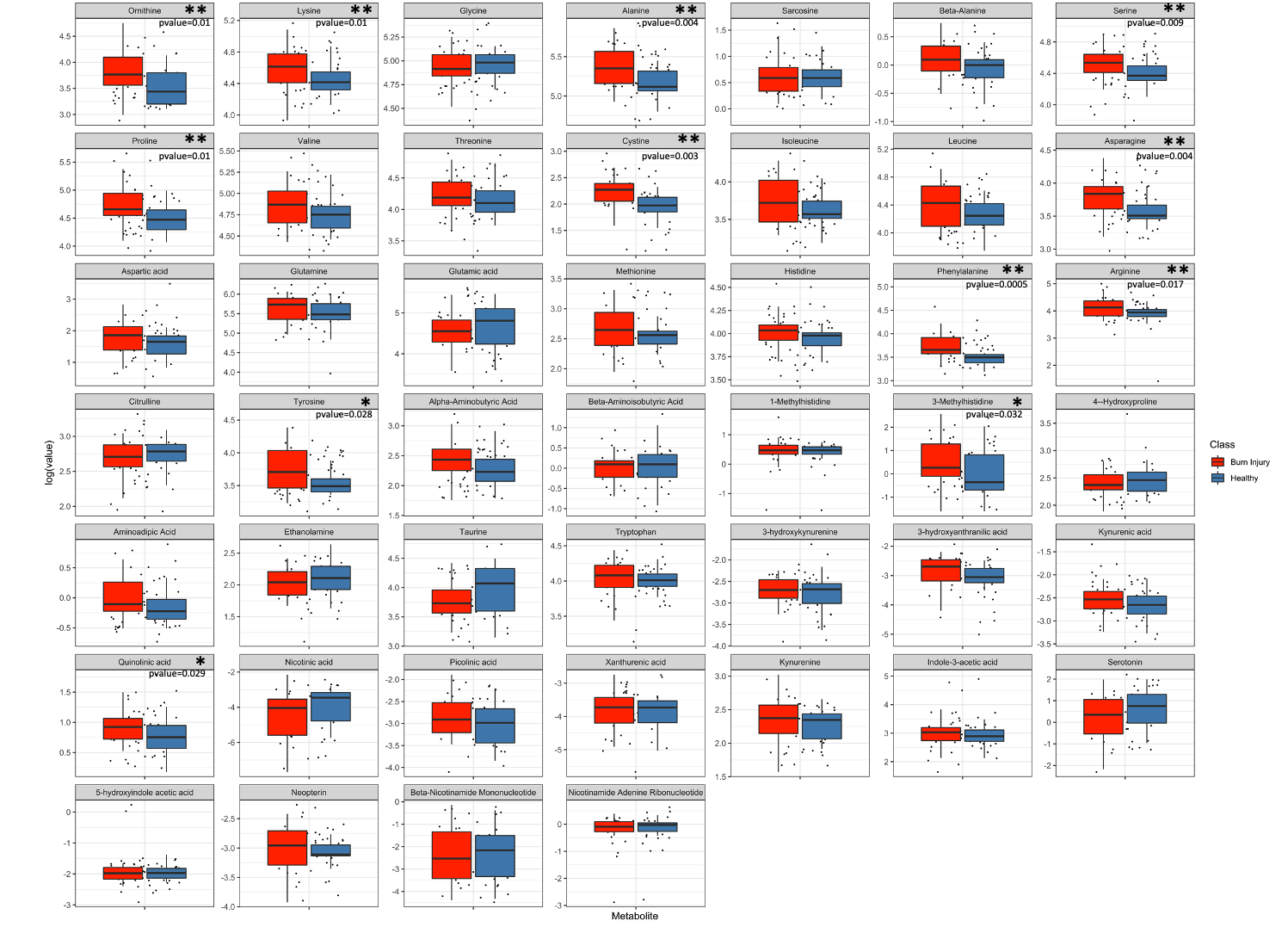


**Figure S5:** A variable importance of projection (VIP) plot of the most significant discriminatory metabolites presents 22 metabolites out of 46 to be class discriminatory i.e. with a value > 1.0: Lysine, Alanine, Tyrosine, Asparagine, Proline, Leucine, Methionine, Serine, Taurine, Aspartic acid, Ornithine, Arginine, Aminoadipic acid, Isoleucine, Histidine, Valine, Serotonin, Ethanolamine, 3-Methylhistidine, Threonine, Cystine and Quinolinic acid.
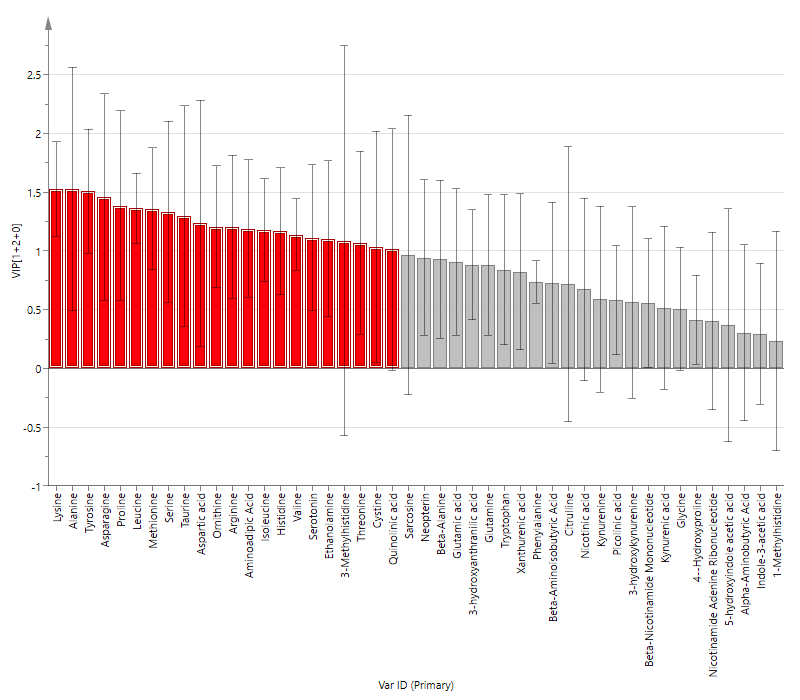


**Table S1:** Univariate analysis of phenotyping panel between burn Injury and non-burn controls (Wilcoxon Rank Sum testing corrected for multiple testing (Benjamini-Hochberg).

| Metabolite | Lower 95% confidence interval | Upper 95% confidence interval | p.value (adjusted) | Effect Size *(r)* | Fold Change | VIP |
| --- | --- | --- | --- | --- | --- | --- |
| Phenylalanine | 2.800E+00 | 1.260E+01 | 0.001** | 7.000E+00 | 7.023E-01 | 5.790E-01 |
| Cystine | 7.999E-01 | 3.500E+00 | 0.003** | 2.200E+00 | 8.544E-01 | 1.052E+00 |
| Asparagine | 3.000E+00 | 1.470E+01 | 0.004** | 9.300E+00 | 8.678E-01 | 1.507E+00 |
| Alanine | 1.080E+01 | 6.890E+01 | 0.004** | 4.180E+01 | 8.748E-01 | 1.572E+00 |
| Serine | 3.300E+00 | 2.060E+01 | 0.009** | 1.196E+01 | 7.405E-01 | 1.344E+00 |
| Proline | 5.000E+00 | 3.970E+01 | 0.010** | 2.386E+01 | 9.376E-01 | 1.412E+00 |
| Lysine | 3.000E+00 | 2.800E+01 | 0.016** | 1.597E+01 | 7.756E-01 | 1.531E+00 |
| Arginine | 1.800E+00 | 2.320E+01 | 0.017** | 1.211E+01 | 9.047E-01 | 1.181E+00 |
| Ornithine | 3.300E+00 | 1.870E+01 | 0.018** | 1.110E+01 | 1.025E+00 | 1.246E+00 |
| Tyrosine | 4.000E-01 | 1.730E+01 | 0.028* | 5.700E+00 | 9.288E-01 | 1.533E+00 |
| Quinolinic acid | 1.742E-02 | 7.373E-01 | 0.029* | 3.842E-01 | 8.084E-01 | 1.017E+00 |
| 3-Methylhistidine | 9.995E-02 | 1.700E+00 | 0.032* | 5.999E-01 | 1.624E+00 | 1.124E+00 |
| Histidine | -3.000E-01 | 7.900E+00 | 0.069 | 3.400E+00 | 6.127E-01 | 1.109E+00 |
| Aspartic acid | -1.000E-01 | 2.700E+00 | 0.071 | 1.300E+00 | 1.178E+00 | 1.253E+00 |
| 3-hydroxyanthranilic acid | -1.143E-03 | 2.916E-02 | 0.072 | 1.465E-02 | 8.787E-01 | 8.090E-01 |
| Aminoadipic Acid | -7.457E-05 | 3.000E-01 | 0.092 | 1.000E-01 | 8.670E-01 | 1.142E+00 |
| Taurine | -2.350E+01 | 2.200E+00 | 0.096 | -1.146E+01 | 2.291E-01 | 1.317E+00 |
| Nicotinic acid | -1.914E-02 | 2.185E-03 | 0.101 | -6.673E-03 | 1.944E-01 | 6.960E-01 |
| Leucine | -1.600E+00 | 2.510E+01 | 0.103 | 1.203E+01 | 7.910E-01 | 1.351E+00 |
| Valine | -2.900E+00 | 2.690E+01 | 0.105 | 1.140E+01 | 6.795E-01 | 1.122E+00 |
| Alpha-Aminobutyric Acid | -4.000E-01 | 3.300E+00 | 0.106 | 1.700E+00 | 6.571E-01 | 3.440E-01 |
| Methionine | -4.000E-01 | 4.500E+00 | 0.149 | 1.600E+00 | 8.032E-01 | 1.362E+00 |
| Serotonin | -1.433E+00 | 3.163E-01 | 0.168 | -5.326E-01 | 1.096E-01 | 1.114E+00 |
| Threonine | -3.200E+00 | 1.570E+01 | 0.192 | 6.259E+00 | 6.447E-01 | 1.011E+00 |
| Picolinic acid | -6.653E-03 | 2.393E-02 | 0.221 | 1.010E-02 | 8.212E-01 | 3.980E-01 |
| Ethanolamine | -1.900E+00 | 4.000E-01 | 0.236 | -8.000E-01 | 3.438E-01 | 1.115E+00 |
| Beta-Alanine | -9.996E-02 | 3.001E-01 | 0.237 | 1.001E-01 | 7.570E-01 | 8.300E-01 |
| Isoleucine | -2.200E+00 | 1.350E+01 | 0.250 | 4.900E+00 | 7.676E-01 | 1.152E+00 |
| Tryptophan | -3.275E+00 | 1.069E+01 | 0.270 | 3.645E+00 | 5.773E-01 | 7.960E-01 |
| Glutamine | -1.750E+01 | 8.510E+01 | 0.283 | 3.445E+01 | 6.444E-01 | 8.730E-01 |
| Glutamic acid | -5.010E+01 | 1.670E+01 | 0.332 | -1.494E+01 | 3.532E-01 | 9.110E-01 |
| Neopterin | -5.182E-03 | 1.377E-02 | 0.389 | 4.726E-03 | 6.758E-01 | 9.910E-01 |
| Citrulline | -2.700E+00 | 1.300E+00 | 0.400 | -8.000E-01 | 4.443E-01 | 7.730E-01 |
| Kynurenic acid | -7.072E-03 | 2.046E-02 | 0.408 | 6.422E-03 | 6.928E-01 | 5.070E-01 |
| Xanthurenic acid | -4.211E-03 | 8.986E-03 | 0.512 | 1.969E-03 | 8.239E-01 | 7.970E-01 |
| Beta-Aminoisobutyric Acid | -3.001E-01 | 1.001E-01 | 0.517 | -9.996E-02 | 3.452E-01 | 8.010E-01 |
| Glycine | -1.630E+01 | 8.100E+00 | 0.553 | -4.400E+00 | 4.731E-01 | 5.090E-01 |
| Indole-3-acetic acid | -3.004E+00 | 5.414E+00 | 0.556 | 1.319E+00 | 4.803E-01 | 2.690E-01 |
| Kynurenine | -1.199E+00 | 2.058E+00 | 0.580 | 4.253E-01 | 5.886E-01 | 6.090E-01 |
| 1-Methylhistidine | -2.000E-01 | 2.999E-01 | 0.628 | 1.631E-05 | 4.229E-01 | 2.450E-01 |
| 4--Hydroxyproline | -1.900E+00 | 1.100E+00 | 0.643 | -3.999E-01 | 5.083E-01 | 4.070E-01 |
| 5-hydroxyindole acetic acid | -2.539E-02 | 1.563E-02 | 0.664 | -5.672E-03 | 9.402E-01 | 3.940E-01 |
| Beta-Nicotinamide Mononucleotide | -7.920E-02 | 4.278E-02 | 0.676 | -7.023E-03 | 6.177E-01 | 5.120E-01 |
| 3-hydroxykynurenine | -1.021E-02 | 1.533E-02 | 0.792 | 2.003E-03 | 5.722E-01 | 6.090E-01 |
| Sarcosine | -2.999E-01 | 4.000E-01 | 0.884 | 8.132E-05 | 6.667E-01 | 1.044E+00 |
| Nicotinamide Adenine Ribonucleotide | -1.599E-01 | 1.348E-01 | 0.939 | -6.049E-03 | 5.313E-01 | 3.780E-01 |

*** pvalue <0.05, **pvalue <0.01.**

**Table S2:** Calculated metabolite ratios between Burn Injury and Healthy Controls

| Ratios | Burn Injury | Healthy Control | Fisher’s exact test |
| --- | --- | --- | --- |
| Glutamine/Glutamate | 2.64:1  [0.14-14.57] | 2.17:1  [0.53-8.69] | 0.16 |
| Tryptophan/Kynurenine | 5.48:1  [3.42-8.79] | 5.53:1  [4.11-9.26] | 0.45 |
| Serotonin /5-Hydroxyindole acetic acid | 10.16:1  [0.33-43.56] | 17.4:1  [2.62-52.57] | 0.11 |
| Phenylalanine/Tyrosine | 0.97:1  [0.61-1.4] | 1.1:1  [0.55-5.50] | 0.21 |
| Quinolinic Acid/Kynurenic Acid | 31.37:1  [10.38-83.53] | 29.54:1  [17.94-52.83] | 0.11 |
| 3-hydroxykynurenine/Kynurenic Acid | 0.82:1  [0.32-1.97] | 0.89:1  [0.45-1.62] | 0.27 |
| Quinolinic Acid/Tryptophan | 0.05:1  [0.02-0.10] | 0.039:1  [0.01-0.06] | 0.02* |
| Fischer's Ratio | 1.95:1  [1.94-4.68] | 2.05:1  [1.25-4.88] | 0.07 |

***Fisher’s exact pvalue <0.05**

**Table S3: Significant metabolite-cytokine correlations ranked by p value (<0.05) in burn injury**

| Metabolite | Cytokine | Spearmans Rho | P value |
| --- | --- | --- | --- |
| Glutamine | IL-6 | -0.542364532 | 0.00236995 |
| Citrulline | IFNg | -0.52944802 | 0.00314219 |
| Kynurenic acid | IL-2 | -0.508190668 | 0.00488349 |
| Threonine | GM-CSF | 0.505480974 | 0.00515562 |
| Aspartic acid | IL-13 | 0.491993117 | 0.00671036 |
| Aspartic acid | IFNg | 0.47874831 | 0.00860661 |
| Citrulline | IL-17A | -0.474741266 | 0.00926232 |
| Cystine | IL-8 | 0.462921916 | 0.01144602 |
| Isoleucine | GM-CSF | 0.459169852 | 0.01222336 |
| Aspartic acid | IL-6 | 0.455530933 | 0.01301889 |
| 3-hydroxykynurenine:Kynurenic Acid | IFNg | 0.4505481 | 0.01417784 |
| Glutamine:Glutamic acid | IL-6 | -0.448275862 | 0.01473413 |
| 3-hydroxykynurenine:Kynurenic Acid | IL-8 | 0.442857143 | 0.01613426 |
| Taurine | IL-1B | 0.442665356 | 0.01618577 |
| Aspartic acid | IL-17A | 0.433411362 | 0.01883799 |
| Glutamic acid | IL-6 | 0.42911689 | 0.0201852 |
| Ethanolamine | GM-CSF | 0.423816774 | 0.02195614 |
| Alpha-Aminobutyric Acid | IL-10 | -0.418587532 | 0.02382632 |
| Methionine | GM-CSF | 0.416163636 | 0.02473633 |
| Phenylalanine:Tyrosine | IL-12(p70) | -0.413597737 | 0.02573038 |
| Aspartic acid | IL-12(p70) | 0.413206853 | 0.02588463 |
| Serine | GM-CSF | 0.411235706 | 0.02667401 |
| Tyrosine | GM-CSF | 0.408424686 | 0.02783354 |
| Lysine | GM-CSF | 0.408374384 | 0.02785465 |
| Citrulline | IL-1B | -0.408329239 | 0.02787361 |
| Tryptophan:Kynurenine | GM-CSF | 0.405418719 | 0.02911846 |
| Valine | GM-CSF | 0.403940887 | 0.02976759 |
| Serine | IL-7 | 0.402045355 | 0.03061731 |
| Taurine | IL-13 | 0.401477833 | 0.0308755 |
| 3-hydroxykynurenine:Kynurenic Acid | IL-10 | 0.398817591 | 0.03210935 |
| Asparagine | IL-8 | 0.398522167 | 0.03224879 |
| Glutamine | TNFa | -0.396551724 | 0.03319143 |
| Taurine | IL-8 | 0.396059113 | 0.03343053 |
| Glutamine | IL-13 | -0.395566502 | 0.03367101 |
| Lysine | IL-8 | 0.393596059 | 0.03464694 |
| 3-hydroxykynurenine | TNFa | 0.38817734 | 0.03744864 |
| Neopterin | IL-8 | 0.387684729 | 0.03771209 |
| Beta-Alanine | IL-10 | 0.387518681 | 0.03780123 |
| Aspartic acid | IL-7 | 0.386842432 | 0.03816601 |
| Taurine | GM-CSF | 0.386699507 | 0.03824347 |
| Taurine | IL-5 | 0.382758621 | 0.04042926 |
| 3-hydroxykynurenine:Kynurenic Acid | IL-17A | 0.378618059 | 0.04283224 |
| 3-hydroxykynurenine:Kynurenic Acid | IL-1B | 0.378371724 | 0.04297871 |
| Neopterin | TNFa | 0.378325123 | 0.04300646 |
| Glutamic acid | IL-13 | 0.377879052 | 0.04327285 |
| Aminoadipic Acid | GM-CSF | 0.376523327 | 0.04409054 |
| 3-hydroxykynurenine:Kynurenic Acid | IL-2 | 0.375908366 | 0.04446548 |
| Aspartic acid | IL-5 | 0.375461948 | 0.04473924 |
| 3-Methylhistidine | TNFa | -0.373998261 | 0.04564626 |
| Fischer's Ratio | IL-10 | -0.36753295 | 0.04982885 |

**Table S4: Significant metabolite-cytokine correlations ranked by p value (<0.05) in non-burn “healthy” controls.**

| Metabolite | Cytokine | Spearman's Rho | P Value |
| --- | --- | --- | --- |
| Asparagine | GM-CSF | -0.7145071 | 0.00086317 |
| Lysine | IL-5 | -0.6525556 | 0.00333033 |
| Isoleucine | IL-1B | -0.6078431 | 0.00745005 |
| Proline | IL-7 | 0.60609198 | 0.00767065 |
| 4--Hydroxyproline | IFNg | -0.6019619 | 0.00821186 |
| Lysine | IL-1B | -0.5926692 | 0.00954244 |
| Lysine | IL-13 | -0.5875065 | 0.01035327 |
| Lysine | TNFa | -0.586474 | 0.01052187 |
| Serine | IL-5 | -0.5596284 | 0.01573675 |
| Leucine | IL-1B | -0.5562436 | 0.0165184 |
| 1-Methylhistidine | IFNg | -0.5506619 | 0.01787418 |
| Asparagine | IFNg | -0.5472381 | 0.01874833 |
| Lysine | IL-6 | -0.5296852 | 0.02377242 |
| Asparagine | IL-1B | -0.5049046 | 0.03258924 |
| Beta-Nicotinamide Mononucleotide | GM-CSF | 0.49019608 | 0.03890345 |
| Kynurenic acid | IFNg | -0.4901961 | 0.03890345 |
| Asparagine | TNFa | -0.4863191 | 0.04071408 |
| Isoleucine | IL-2 | -0.4840041 | 0.04182552 |
| Asparagine | IL-5 | -0.4811565 | 0.04322426 |
| Picolinic acid | IL-2 | -0.4798762 | 0.04386464 |
| 3-Methylhistidine | IL-6 | 0.47432261 | 0.04672629 |
| Isoleucine | IL-5 | -0.4695562 | 0.04929338 |

**Table S5: List of metabolites from quantitative enrichment analysis for the top 25 pathway hits.**

| Metabolite Set | P value | FDR | Metabolites |
| --- | --- | --- | --- |
| Selenoamino Acid Metabolism | 0.002046 | 0.121 | Alanine, Nicotinamide Adenine Ribonucleotide |
| Catecholamine Biosynthesis | 0.006332 | 0.121 | Tyrosine |
| Thyroid hormone synthesis | 0.006332 | 0.121 | Tyrosine |
| Glycine and Serine Metabolism | 0.00911 | 0.121 | Glycine, Glutamic acid, Alanine, Threonine, Ornithine,  Sarcosine, Arginine, Methionine, Nicotinamide Adenine Ribonucleotide |
| Spermidine and Spermine Biosynthesis | 0.011936 | 0.121 | Ornithine, Methionine |
| Glucose-Alanine Cycle | 0.012429 | 0.121 | Glutamic acid, Alanine, Nicotinamide Adenine Ribonucleotide |
| Glutathione Metabolism | 0.014319 | 0.121 | Glycine, Glutamic acid, Alanine |
| Alanine Metabolism | 0.014319 | 0.121 | Glycine, Glutamic acid, Alanine |
| Tryptophan Metabolism | 0.014328 | 0.121 | Glutamic acid, Alanine, Nicotinamide Adenine Ribonucleotide, Indoleacetic acid, Quinolinic acid, Serotonin, Kynurenine,  Kynurenic acid,  5-Hydroxyindoleacetic acid, Xanthurenic acid, Tryptophan, 3-Hydroxyanthranilic acid |
| Biotin Metabolism | 0.018277 | 0.1389 | Lysine |
| Betaine Metabolism | 0.031375 | 0.21677 | Methionine, Nicotinamide Adenine Ribonucleotide |
| Taurine and Hypotaurine Metabolism | 0.043841 | 0.27252 | Taurine |
| Carnitine Synthesis | 0.046615 | 0.27252 | Glycine, Lysine, Nicotinamide Adenine Ribonucleotide |
| Methylhistidine Metabolism | 0.056245 | 0.29785 | Histidine, 3-Methylhistidine |
| Arginine and Proline Metabolism | 0.058786 | 0.29785 | Glycine, Glutamic acid, Proline, Aspartic acid, Ornithine, Arginine, Citrulline, Nicotinamide Adenine Ribonucleotide |
| Urea Cycle | 0.066547 | 0.3161 | Glutamic acid, Alanine, Aspartic acid, Ornithine,  Arginine, Glutamine,  Citrulline, Nicotinamide Adenine Ribonucleotide |
| Glutamate Metabolism | 0.085912 | 0.38408 | Glutamic acid, Alanine, Aspartic acid, Glycine,  Arginine, Glutamine, Nicotinamide Adenine Ribonucleotide |
| Phosphatidylcholine Biosynthesis | 0.12412 | 0.47899 | Ethanolamine |
| Phosphatidylethanolamine Biosynthesis | 0.12412 | 0.47899 | Ethanolamine |
| Phospholipid Biosynthesis | 0.12605 | 0.47899 | Ethanolamine, Nicotinamide Adenine Ribonucleotide |
| Bile Acid Biosynthesis | 0.16806 | 0.60821 | Nicotinamide Adenine Ribonucleotide, Glycine, Taurine |
| Valine, Leucine and Isoleucine Degradation | 0.21797 | 0.753 | Nicotinamide Adenine Ribonucleotide, Glutamic acid, Isoleucine. Leucine, Valine |
| Threonine and 2-Oxobutanoate Degradation | 0.23895 | 0.78956 | Threonine, Nicotinamide Adenine Ribonucleotide |
| Lysine Degradation | 0.26687 | 0.81087 | Glutamic acid, Lysine, Aminoadipic acid, Nicotinamide Adenine Ribonucleotide |
| Aspartate Metabolism | 0.27705 | 0.81087 | Beta-Alanine, Glutamic acid, Asparagine, Aspartic Acid, Arginine, Glutamine, Citrulline |

**Table S6:** Demographic information of recruited pediatric burn patients

| **Age at collection/ years** | **Gender** | **Age at time of burn/months** | **TBSA %** | **Etiology of injury** |
| --- | --- | --- | --- | --- |
| 4 | f | 37 | 3% | scald |
| 5 | f | 13 | 7% | scald |
| 5 | f | 18 | 3% | scald |
| 5 | f | 18 | 2% | frictional |
| 5 | f | 25 | 8% | scald |
| 5 | f | 12 | 6% | scald |
| 5 | f | 24 | 1% | Frictional |
| 5 | m | 24 | 10% | scald |
| 5 | m | 12 | 1% | Scald |
| 5 | m | 12 | 5% | Contact |
| 5 | m | 16 | 2% | Contact |
| 6 | f | 22 | 1% | frictional |
| 6 | f | 25 | 2.50% | scald |
| 6 | f | 18 | 1.50% | scald |
| 6 | f | 12 | 7% | scald |
| 6 | f | 19 | 5% | Scald |
| 6 | f | 12 | 2% | chemical |
| 6 | f | 10 | 1% | Contact |
| 6 | m | 15 | 3% | scald |
| 6 | m | 18 | 9% | scald |
| 6 | m | 7 | 1% | electrical |
| 6 | m | 12 | 2% | Scald |
| 6 | m | 18 | 1% | Contact |
| 6 | m | 6 | 10% | Sun burn |
| 6 | m | 12 | 8% | Scald |
| 6 | m | 18 | 9% | Scald |
| 6 | m | 18 | 2% | Thermal |
| 6 | m | 18 | 5% | chemical |
| 6 | m | 30 | 2% | contact |
| 7 | f | 14 | 3% | Contact |
| 7 | m | 41 | 1% | frictional |
| 8 | m | 38 | 10% | Contact |
| 8 | m | 38 | 1% | Scald |

**Table S7:**

Lower limits of quantification (LLOQ) for each metabolite targeted in the liquid chromatography-mass spectrometry (LC-MS) analysis

| **Metabolite** | **LLOQ (nmol/L)** |
| --- | --- |
| Phenylalanine | 1000 |
| Cystine | 1000 |
| Asparagine | 1000 |
| Alanine | 1000 |
| Serine | 1000 |
| Proline | 1000 |
| Lysine | 1000 |
| Arginine | 1000 |
| Ornithine | 1000 |
| Tyrosine | 1000 |
| Quinolinic acid | 12.0 |
| 3-Methylhistidine | 1000 |
| Histidine | 1000 |
| Aspartic acid | 1000 |
| 3-hydroxyanthranilic acid | 13.1 |
| Aminoadipic Acid | 1000 |
| Taurine | 1000 |
| Nicotinic acid | 3.25 |
| Leucine | 1000 |
| Valine | 1000 |
| Alpha-Aminobutyric Acid | 1000 |
| Methionine | 1000 |
| Serotonin | 1000 |
| Threonine | 1000 |
| Picolinic acid | 3.25 |
| Ethanolamine | 1000 |
| Beta-Alanine | 1000 |
| Isoleucine | 1000 |
| Tryptophan | 979 |
| Glutamine | 1000 |
| Glutamic acid | 1000 |
| Neopterin | 0.79 |
| Citrulline | 1000 |
| Kynurenic acid | 2.11 |
| Xanthurenic acid | 1.95 |
| Beta-Aminoisobutyric Acid | 1000 |
| Glycine | 1000 |
| Indole-3-acetic acid | 114 |
| Kynurenine | 19.2 |
| 1-Methylhistidine | 1000 |
| 4-Hydroxyproline | 1000 |
| 5-hydroxyindole acetic acid | 10.5 |
| Beta-Nicotinamide Mononucleotide | 12.0 |
| 3-hydroxykynurenine | 8.92 |
| Sarcosine | 1000 |
| Nicotinamide Adenine Ribonucleotide | 2.99 |
